# The Minichromosome Maintenance Complex Component 2 *(MjMCM2)* of *Meloidogyne javanica* is a potential effector regulating the cell cycle in nematode-induced galls

**DOI:** 10.1038/s41598-022-13020-8

**Published:** 2022-06-02

**Authors:** Nathalia Fitoussi, Janice de Almeida Engler, Natalia Sichov, Patricia Bucki, Noa Sela, Arye Harel, Eduard Belausuv, Anil Kumar, Sigal Brown Miyara

**Affiliations:** 1 Department of Entomology, Nematology and Chemistry Units, Agricultural Research Organization (ARO), The Volcani Center, 50250 Bet Dagan, Israel; 2 Department of Plant Pathology and Microbiology, The Faculty of Agriculture Food and Environment, The Hebrew University of Jerusalem, 76100 Rehovot, Israel; 3 INRAE, CNRS, ISA, Université Côte d’Azur, 06903 Sophia Antipolis, France; 4 Bioinformatics Unit, Institute of Plant Sciences, Agricultural Research Organization (ARO), The Volcani Center, 50250 Bet Dagan, Israel; 5 Department of Plant Sciences, Agricultural Research Organization (ARO), The Volcani Center, Bet Dagan, Israel

**Keywords:** Molecular biology, Plant sciences, Plant signalling, Plant stress responses

## Abstract

Root-knot nematodes *Meloidogyne* spp. induce enlarged multinucleate feeding cells—galls—in host plant roots. Although core cell-cycle components in galls follow a conserved track, they can also be usurped and manipulated by nematodes. We identified a candidate effector in *Meloidogyne javanica* that is directly involved in cell-cycle manipulation—Minichromosome Maintenance Complex Component 2 (MCM2), part of MCM complex licensing factor involved in DNA replication. *MjMCM2*, which is induced by plant oxilipin 9-HOT, was expressed in nematode esophageal glands, upregulated during parasitic stages, and was localized to plant cell nucleus and plasma membrane. Infected tomato hairy roots overexpressing *MjMCM2* showed significantly more galls and egg-mass-producing females than wild-type roots, and feeding cells showed more nuclei. Phylogenetic analysis suggested seven homologues of MjMCM2 with unknown association to parasitism. Sequence mining revealed two RxLR-like motifs followed by SEED domains in all *Meloidogyne* spp. MCM2 protein sequences. The unique second RxLR-like motif was absent in other *Tylenchida* species. Molecular homology modeling of MjMCM2 suggested that second RxLR2-like domain is positioned on a surface loop structure, supporting its function in polar interactions. Our findings reveal a first candidate cell-cycle gene effector in *M. javanica*—MjMCM2—that is likely secreted into plant host to mimic function of endogenous MCM2.

## Introduction

Some of the most damaging plant-parasitic nematodes (PPNs) are the *Meloidogyne* spp. root-knot nematodes (RKNs), an obligatory sedentary endoparasite infecting a wide range of hosts^1,2^. The RKN species are known for their ability to manipulate the morphology and physiology of their host, facilitating the establishment of a long-term association. Motile *Meloidogyne* spp. second-stage juveniles (J2s) penetrate the host roots and migrate intercellularly to reach parenchyma cells in the vascular cylinder that display competence to reenter the cell cycle^3^. Cells pierced by juvenile nematodes are then induced to undergo several rounds of mitotic nuclear division without cytokinesis, followed by recurring cycles of DNA replication, leading to the formation of multiple aberrant giant cells (GCs) that serve as the feeding site for RKN survival^4,5^. In addition to the multiple enlarged nuclei, these reprogrammed GCs are characterized by invaginated cell walls, and a dense cytoplasm containing small vacuoles replacing the large central vacuole, reflecting the metabolic activity needed for nematode growth and reproduction^6,7^. During gall formation and development, plant host cell cycle-regulating genes are differentially expressed, and they have been functionally analyzed. Examples are the core cell-cycle genes *CDKA1*, *CDKB1,1*, *CYCB1,1*, *CYCA2,1*, and others involved in DNA replication, such as *ORC1-6*, *MCM5* and *CDC6,* the endocycle activators *CCS52A1–2*, *CCS52B,* and genes involved in the cell-cycle control of galls such as *DEL1, KRP1-7, WEE1* and *ABAP1*^8–15^. In addition, hormone metabolism is differentially regulated in GCs, with high concentrations of auxins and cytokinins in the galls^16,17^. This successful plant–nematode interaction relies on the secretion of effectors produced mainly in the nematode's esophageal glands and injected into the host root cell that is competent to reenter the cell cycle^11,18–23^.

The last decades have seen attempts to elucidate the host mechanisms that are manipulated by effectors in GCs during their development and functioning. Recently, effectors involved in promoting susceptibility or resistance, penetration, cell wall degradation, and cytoskeletal rearrangements have been reviewed by Vieira and Gleason^24^. However, no effectors that are directly involved in host cell-cycle manipulation, by triggering or mimicking plant cell-cycle regulators, have been identified to date. Previously we show that exposure of juvenile *Meloidogyne javanica* to the oxylipin 9-hydroxyoctadecatrienoic acid (9-HOT), a product of the plant host's 9-lipoxygenase pathway, induces upregulation of Minichromosome Maintenance Protein Subunit 2 (MCM2)^25^. MCM2 is a part of the protein complex MCM2–7 that plays a crucial role in cell division, acting as a licensing factor for DNA replication, as well as for transcription and replication checkpoints and RNA splicing^26–28^. In plants, MCM complex proteins have been found to be highly expressed in proliferating tissues such as the root tips and shoot apex, and to play a role in the abiotic stress response^29,30^. Furthermore, upregulation of the six MCM proteins of the MCM2–7 family in plant cells was found to activate the cell cycle^31^.

In the current study, we show that *MjMCM2* is expressed during parasitism and is particularly localized within *M. javanica* J2 esophageal glands, demonstrating the two main criteria for its consideration as a candidate effector. Further, phylogenetic analysis suggested that *M. javanica* harbors seven homologues of *MjMCM2*, whose association with parasitism has yet to be revealed. While no canonical signal peptide was found following the *in silico* analysis, two inlaid RxLR-like motifs, were revealed along the MjMCM2 protein, with the second RxLR-like motif found at the C terminus and only conserved in *Meloidogyne* spp. Taken together, we hypothesize that MjMCM2 is a candidate effector protein of *M. javanica* that acts as a cell-cycle regulator to facilitate the manipulation of vascular parenchyma cells to induce GC genesis.

## Materials and methods

### Nematode growth, extraction and sterilization of eggs

*Meloidogyne javanica* was propagated for 4–6 weeks on tomato plants (*Solanum lypopersicum* cv. Avigail 870) grown in a glasshouse under a 16 h:8 h, light:dark photoperiod at 25 °C. Roots were washed and cut into segments, macerated in 0.05% (v/v) sodium hypochlorite (NaOCl) in a Waring Commercial Blender, 800G, at 22,000 rpm for 3 min, and subjected to centrifugal flotation as described by Hussey^20^ to extract the nematodes eggs. The supernatant, containing the eggs, was poured onto a 30-µm sieve, and the eggs were washed with tap water and collected in 0.01 M MES buffer (Sigma-Aldrich, St. Louis, US). Nematode eggs were sterilized as described by Jansen van Vuuren and Woodward^32^, then collected and transferred onto a 30-µm sieve in a petri dish with 5 ml 0.01 M MES buffer. The petri dish was then placed in a growth chamber at 26 °C under dark conditions till hatching (5–6 days). All described experimental research on plants material was conducted under institutional and international guidelines and legislations.

### Total RNA extraction from five *M. javanica* developmental stages

*M. javanica* eggs, freshly hatched preparasitic J2s (ppJ2s), parasitic J2s (pJ2s) 12 h after inoculation, three- to four-stage juveniles (J3–4s) and mature females were collected for total RNA extraction. The eggs and ppJ2s were collected right after sterilization. All other parasitic stages were isolated from the roots of *in vitro*-grown plants. Seeds of tomato cv. Avigail 870 were sterilized by soaking in 1.4% NaOCl for 10 min, washed three times with sterile water for 5 min, and then plated on standard-strength Gambourg's B5 medium salt mixture (Duchefa, Haarlem; The Netherlands), supplemented with 2% (w/v) sucrose and solidified with 0.8% (w/v) Gelrite agar (Duchefa) as described earlier by Iberkleid et al.^33^. Seeds were kept in a growth chamber at 26 °C for 3 d in the dark, and then transferred to a 16 h:8 h, light:dark photoperiod (120 µmol m^−2^ s^−1^). Two weeks after germination, tomato root segments were subcultured by placing one root piece per new Petri dish (90mm) (Miniplast, M.P. Hefer, Israel) containing Gambourg’s B5 medium salt mixture for an additional week at 26 °C under dark conditions before nematode inoculation. Plates containing tomato roots were inoculated with 300 sterile ppJ2s; 12 h later, the typical thick hairy areas of root material (0.5–1 cm roots tissues), indicating nematode penetration, were collected into a 1.5-ml tube (~ 50 mg). For later time points, galls were collected 15 days after inoculation (DAI) for J2s and J3–4 stages, and 28 DAI for females without egg masses (harvesting ~ 50 mg root tissues for each stage and time point). RNA extracted from uninfected roots was used as a negative control. All samples were immersed in liquid nitrogen and stored at − 80 °C before RNA isolation.

### Real-time quantitative PCR analysis

The mRNA was extracted from all *M. javanica* developmental stages and non-inoculated tomato roots with Invitrogen TRIzol^TM^ reagent (Thermo Fisher Scientific, Carlsbad, CA, US) and cDNA was synthesized by Verso cDNA synthesis kit (Thermo Fisher Scientific), both according to the manufacturer's instructions. The putative effector *MjMCM2* was amplified with the forward primer: 5'-CTGACTCTTTAACTGACGAAGAC-3' and reverse primer: 5'-GCAATACTGGCAAAAATTCGTTG-3' according to MjMCM2 accession M.Javanica_Scaff2271g021798. For all real-time quantitative PCR (qRT-PCR) analyses, two housekeeping genes were chosen as reference genes for *M. javanica*: endogenous reference genes *18S* (GenBank Accession No. BH012957.1) and *EF-1α* (GenBank Accession No. U94493.1). Primer design for qRT-PCR was conducted by Primer3 software^34^. The qRT-PCR reactions were performed on a StepOnePlus™ Real-Time PCR system (Applied Biosystems, Thermo Fischer Scientific; Carlsbad, CA, US) with the following cyclic conditions: initial heating temperature of 95 °C for 15 min followed by 40 cycles at 95 °C for 15 s, 58 °C for 20 s and 72 °C for 20 s. The PCR products were exposed to melting curve analysis; the conditions were incubation at 60–95 °C with temperature increment of 0.3 °C s^−1^. Each reaction was performed in triplicate and fold changes (FC) of the target genes were calculated by 2^−ΔΔCt^ method^35^ for each treatment compared to the egg treatment, set at FC = 1. Statistical differences between treatments were calculated by least significant difference (LSD) according to Tukey–Kramer multiple comparison test at *P* ≤ 0.05 with JMP Pro 15 software (SAS). Two independent biological experiments were performed.

### Fluorescence *in Situ* Hybridization (FISH) for *MjMCM2* localization in *M. javanica* J2s

Freshly hatched *M. javanica* ppJ2s were treated with 9-HOT diluted in 0.01 M MES buffer to a final concentration of 10 µM, or with 0.01 M MES buffer as a control for 3 h; all samples were washed with 0.01 M MES buffer. The FISH procedure was performed according to Sakurai et al.^36^, with slight adjustments made for nematodes^25^. Fresh ppJ2 nematodes were cut manually with a razor blade and transferred to Carnoy's solution (chloroform:ethanol:glacial acetic acid, 6:3:1, v/v) and fixed overnight. The samples were then cleared in 6% (v/v) hydrogen peroxide in ethanol for 2 h and hybridized overnight in hybridization buffer (20 mM Tris–HCl pH 8.0, 0.9 M NaCl, 0.01% w/v SDS, 30% v/v formamide) containing 10 pmol ml^−1^ fluorescent probe. Based on the sequences of interest in *MCM2*, DNA probes were designed using Primer Express 3.0.1 software and checked for specificity using BLASTn (NCBI), MCM2 Cy5 (5'-GGCTGGCATTGTCACTTCTTTA-3′) was used to target *M. javanica* J2s. The stained samples were submerged in hybridization buffer supplemented with 4',6-diamidino-2-phenylindole (DAPI) (0.1 mg ml^−1^ in 1X PBS) and transferred to a slide with liquid blocker, covered, sealed with nail polish, images were taken with a IX81Olympus FluoView500 confocal microscope (Olympus Corporation, Tokyo, Japan). *M. javanica* exposed to 0.01 M MES buffer only was used as a control. For microscopic observation 20 specimens from each treatment (J2's exposed to 9-HOT or Buffer control) were analyzed for MjMCM2 probe localization.

### Subcellular localization of MjMCM2 *in planta* and vector construction

*MjMCM2* was first amplified, using Platinum Taq DNA Polymerase High Fidelity (Thermo Fisher Scientific, Carlsbad, CA, USA). For that, the full-length cDNA sequence of MjMCM2 was amplified using the MjMCM2F (5′-ATGTATGCTATACGAAGTTATTACG-3′) and MjMCM2R (5′ AGCAATTGTTTTGACAAT-3′) primers designed based on the MjMCM2 (accession# M.Javanica_Scaff2271g021798) sequence of M*. javanica.* PCR reaction was performed as follows: heating to 94 °C for 3 min; 35 cycles of 94 °C for 30s, 60 °C for 30s and 72 °C for 2.5 min; followed by a final extension for 2 min. Amplicon was cloned into pGEM-T (Invitrogen, Carlsbad, CA, USA) easy vector for sequencing. *M. javanica* J2s cDNA was used for gene amplification. Using the corresponding amplicon in pGEM-T as a template, *MjMCM2* was amplified, without the stop codon, using Platinum Taq DNA Polymerase High Fidelity (Thermo Fisher Scientific) using forward primer including the attB1adaptor: 5'-GGGGACAAGTTTGTACAAAAAAGCAGGCTATGTATGCTATACGAAGTTATTACG-3'; reverse primer including the attB2adaptor: 5'-GGGGACCACTTTGTACAAGAAAGCTGGGTAGCAATTGTTTTGACAAT-3', generating a 2400-bp long amplicon. The amplicon then was cloned into the Gateway destination vector pDONR221 (Invitrogen, Carlsbad, CA, USA) by using BP Clonase enzyme mix, The pDONR221:MjMCM2 was transferred to the destination vector pK7FWG2,0^37^, resulting in construct pK7FWG2,0:MjMCM2 expressing enhanced green fluorescent protein (eGFP). For more precise localization of the MCM2-fusion protein, we used three organelle markers: mCherry–endoplasmic reticulum (ER-Rb,35S::mCherry-HDEL), mCherry–Golgi (GmMan1-RFP) and mCherry–cytoplasmic membrane (aquaporin PIP2A:RFP) kindly provided by Dr. Einat Sadot (ARO, Volcani Center, Israel)^38^.

### *Nicotiana benthamiana* plant growth and *Agrobacterium tumefaciens* culture preparation

*N. benthamiana* seedlings were grown in a glasshouse at 25 °C, under a photoperiod regime of 16 h:8 h, light:dark for 3–4 weeks, and *A. tumefaciens* strain GV3101 was used for all infiltrations. *A. tumefaciens* was transformed by using the freeze and thaw method^39^. The transformed *A. tumefaciens* was grown over 2 nights at 28 °C, with shaking at 180 rpm in LB medium including 10 mg ml^−1^ rifampicin, 100 ml^−1^ spectinomycin (for the construct pK7FWG2,0:MjMCM2) and 50 ml^-1^ kanamycin (for the organelle marker constructs), to an optical density at 600 nm (OD_600_) of 0.6–1.0. From each culture, 1 ml was transferred to a new 50-ml tube containing 5 ml of LB medium with the same antibiotics, to an OD_600_ of 0.4 (*c.* 4 h). The bacterial culture was then resuspended in 10 ml MMAi medium for 2 h at 28 °C with shaking at180 rpm, followed by centrifugation for 3 min at 1000 g, then resuspended in 1 ml of MMAi for 1 h at room temperature. The abaxial side of *N. benthamiana* leaves was infiltrated with transformed *A. tumefaciens* at a 1:1 ratio of pK7FWG2,0:MjMCM2 and different mCherry organelle markers. Two plants (about 9 leaves) were infiltrated for each experiment and the same experiment was repeated twice. Free-eGFP control (empty pK7FWG2,0) was used for the comparison (Supplementary Fig. S4).

After infiltration, plants were transferred back to the glasshouse. Images were acquired 48 h after infiltration, using a Leica SP8 laser scanning microscope equipped with solid-state lasers with 488 and 552 nm light under a HC PL APO CS2 63X/1.2 water immersion objective (Leica) and Leica Application Suite X software (LASX). GFP and mCherry emission signals were detected with HyD (hybrid) detectors ranging from 500 to 530 nm and 580 to 650 nm, respectively.

### *In planta* overexpression of MjMCM2

To overexpress MjMCM2, *Rhizobium rhizogenes* ATCC 15834 was transformed with pK7FWG2,0:MjMCM2 construct following the protocol described by Ron et al.^40^. Cotyledons of 10-d-old tomato cv. Avigail 870 seedlings were soaked for 2 h in a 50-ml tube containing 5 ml LB with 10 mg ml^-1^ rifampicin and 100 mg ml^−1^ spectinomycin and respective *Rhizobium* strain at optical density of 0.5 at 600 nm as described by Ron et al.^40^. These samples were then dried on autoclaved filter paper and plated on standard-strength Gambourg’s B5 medium salt mixture, supplemented with 2% sucrose and solidified with 0.8% Gelrite containing 50 mg ml^−1^ kanamycin and 300 mg ml^−1^ timentin. The cotyledons were kept in a growth chamber at 26 °C in the dark. Hairy roots emerging from the cotyledons were transferred to Gamborg’s B5 medium containing 0.8% Gelrite and 50 mg ml^−1^ kanamycin as described by Chinnapandi et al.^41^. The presence and expression of transgenic tomato hairy roots was confirmed by genomic PCR and this line was named *MjMCM2*^*OE*^. For that purpose, total genomic DNA was isolated from the MjMCM2- overexpressing tomato roots and control line using cetyltrimethylammonium bromide (CTAB) method described by Goetz et al.^42^. 50 ng DNA was used to confirm MjMCM2 transgenic lines with forward 5′-ATGTATGCTATACGAAGTTATTACG-3′ and reverse 5′ AGCAATTGTTTTGACAAT-3′ primers, which gave 2400-bp amplicon size. PCR reaction was performed as follows: heating to 94 °C for 3 min, 35 cycles of 94 °C for 30s, 60 °C for 30s and 72 °C for 2.5 min,followed by a final extension for 2 min.

### Response to *M. javanica* infection on transgenic roots overexpressing *MjMCM2*

The *MjMCM2*-overexpressing hairy roots were inoculated with 250 sterilized ppJ2s and infected roots were harvested 28 DAI. These roots were stained with acid fuchsin solution (Sigma-Aldrich, St. Louis, US) (17.5 mg acid fuchsin, 500 ml ethanol and 500 ml acetic acid) and galls were dissected under a stereomicroscope (Olympus SZX12, Tokyo; Japan). Galls and females with and without egg masses were counted for 15 culture plates replicates expressing MjMCM2^OE^ under the constitutive CaMV-35S promoter and for control roots. Statistical differences were determined independently for each experiment by all-means comparison using the Tukey–Kramer test at a level of 0.05, with JMP Pro 15 software (SAS). Two independent biological experiments were conducted.

### Morphological analysis of galls on roots overexpressing MjMCM2

A total of 15 galls from each *MjMCM2*^OE^ line and a control line were collected from 5 plates of tomato roots of each line at 28 DAI. These galls were fixed, dehydrated and embedded in Technovit 7100 (Heraeus Kulzer, Germany) according to the manufacturer’s instructions. For sections preparation, embedded tissues were sectioned to semithin (3 µm-thick) sections using an Ultramicrotome (Leica EM UC7, Leica Microsystems Inc., IL, US), with triangular glass knives. Prior observation, sections were stained with DAPI, 1mg/L DAPI (Sigma, St. Louis, MO) in ddH2O for 30 s. Sections were then imaged under an Olympus BX63 microscope using a UV filter.

### Bioinformatics analysis

Classical nuclear-localization signals or known conserved motifs and domains were predicted using Motif Scan (http://myhits.isb-sib.ch/cgi-bin/motif_scan/). Signal peptide sites were predicted using the SignalP 4.1 server (http://www.cbs.dtu.dk/services/SignalP/;^43^. Subcellular location of effectors was predicted using WoLF PSORT (https://www.genscript.com/wolf-psort.html^44^), ChloroP (http://www.cbs.dtu.dk/services/ChloroP/^45^) and LOCALIZER (http://localizer.csiro.au/^46^). Protein parameters were calculated using ProtParam (http://web.expasy.org/protparam/^47^). Protein sequence alignment was performed with Protein BLAST (https://blast.ncbi.nlm.nih.gov/Blast.cgi). For the identification of RxLR patterns, we used the expandable R package *effectR*^48^. To predict the first set of candidate effector proteins, *effectR* searches the open reading frame (ORF) translation file to find sequences that match the motifs of interest. These searches are based on REGEX matching. For the RxLR motif search, we used **1.** the REGEX reported by Haas et al.^49^: ^\w{10,40}\w{1,96}R\wLR\w{1,40}EER,**2.** to find a motif as part of the REGEX search that does not necessarily include a signal peptide and in addition, identifies effectors with the canonical W–Y–L motif found in RxLR proteins^50^, we implemented the custom script : regex.search (seq=ORF, motif = ",**3.** as part of the RGEX search, we used a pattern that searches for the RxLR motif in a greater area of the protein and also includes the modified regular expression pattern from Haas et al.^49^, who used [ED][ED][KR] to simplify the initial REGEX search ^\\w{1,2000}R\\wLR\\w{1,70}[ED][ED][RKT]. The number of proteins found in every interaction can be seen in Supporting Information Table S1.

### 3D modeling of MjMCM2 protein

We generated a model predicting the 3D structure of MjMCM2 using the SWISS-MODEL server^51^, based on the cryogenic electron microscopy (cryo-EM) structure using PDB id 6XTX.1.A as the template (i.e., the structure used to construct the molecular model), and the linear sequence of the MjMCM2 protein. The template has been reported to be a Cdc45–MCM–GINS (CMG) helicase comprising the MCM2 chain (Chain A, corresponding to UniProt sequence id P49736)^52^. A local alignment score threshold (QMEANDisCo) > 0.60 was used to identify large regions of good local alignment (based on personal correspondence with the SWISS-MODEL team and their web manual).

## Results

### *In silico* structural analysis of *MjMCM2* and transcript abundance

**Figure 1:**
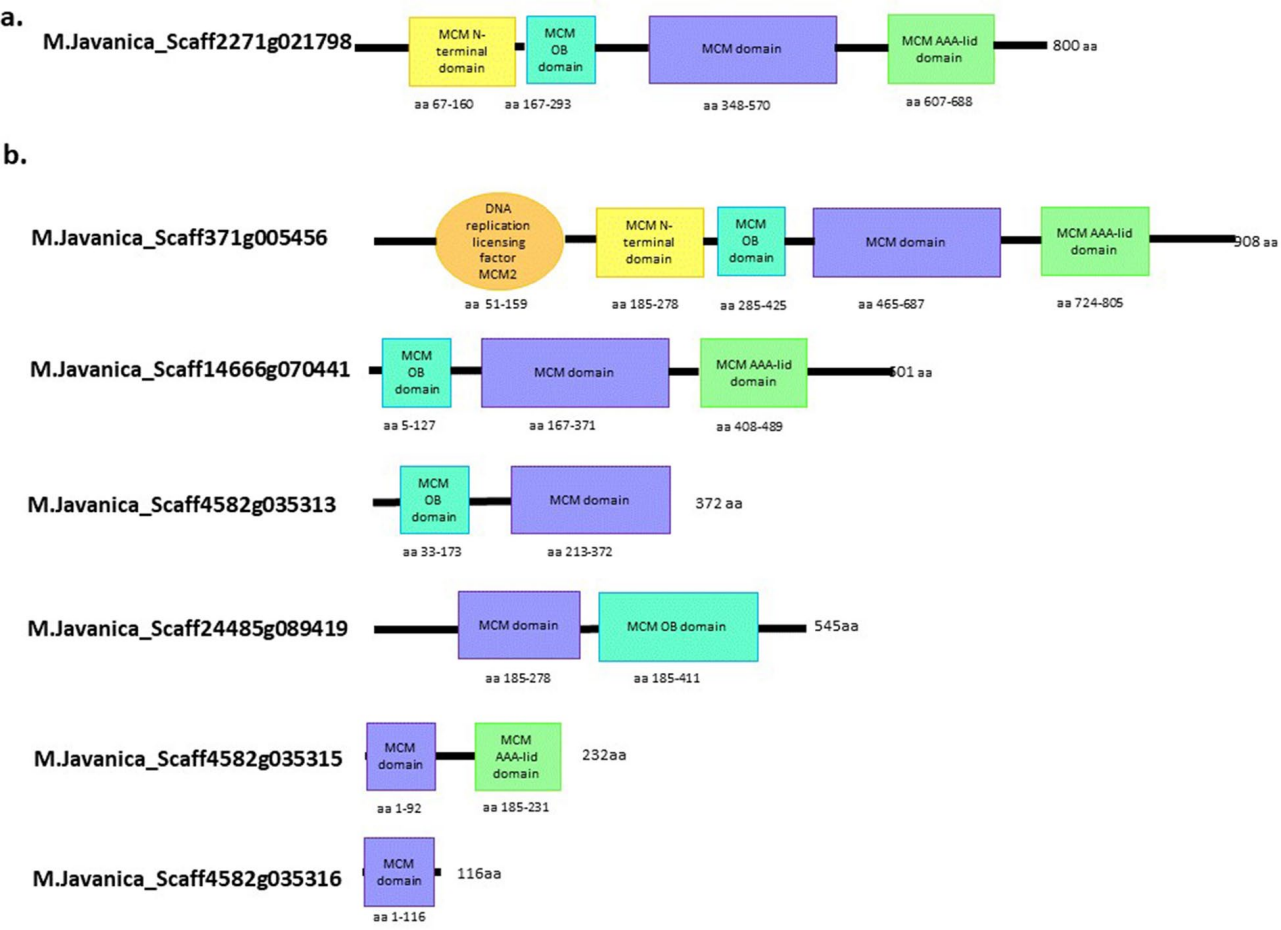
Schematic diagram of overall domain architecture of *Meloidogyne javanica* Minichromosome Maintenance Complex Component 2 (MCM2) amino acid sequences encoded by homologous *MCM2* genes. (**a**) Candidate effector MjMCM2 (*M. Javanica*_Scaff2271g021798). (**b**) All homologous *M. javanica* MCM2 proteins according to WormBase ParaSite^53,54^.

In our previous transcriptomic study^25^, *MjMCM2* (*M.Javanica*_Scaff2271g021798) was found to be upregulated upon exposure of *M. javanica* J2s to the plant oxylipin 9-HOT. The *MjMCM2* cDNA sequence contains 2426 bp encoding an 800-amino acid (aa) protein. This protein carries an MCM N-terminal domain, MCM OB domain, MCM domain and MCM AAA-lid domain, and lacks a classical signal peptide region according to SignalP 5.0 (Fig. 1a). Genome mining and blast analysis revealed six homologous transcripts of *MjMCM2* in *M. javanica*, according to WormBase ParaSite Version WBPS15 (WS276)^53,54^: *M.Javanica*_Scaff4582g035315, *M.Javanica*_Scaff24485g089419, *M.Javanica*_Scaff4582g035313, *M.Javanica*_Scaff4582g035316, *M.Javanica*_Scaff14666g070441 and *M.Javanica*_Scaff371g005456. The latter, longest transcript, contained the most MCM domains, while all other homologous sequences lacked one or more related motifs (Fig. 1b).

### *MjMCM2* expression is induced during nematode parasitic-stage development in tomato roots

**Figure 2:**
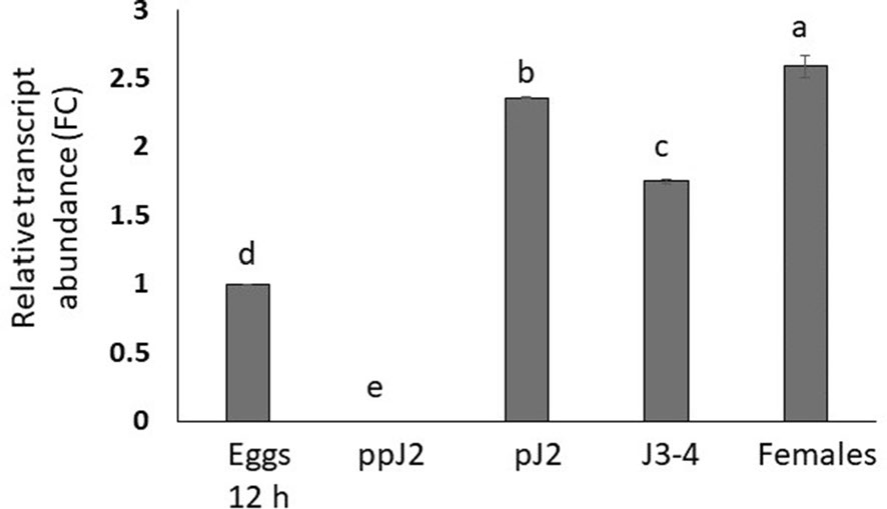
*MjMCM2* expression pattern throughout *M. javanica*'s life cycle. *MjMCM2* transcripts from five different nematode developmental stages (eggs, preparasitic (pp) J2s, parasitic (p) J2s, J3–4s, and mature females) were subjected to qRT-PCR analysis. The transcripts were normalized against two endogenous nematode reference genes, *18S* and *EF-1α*. Each reaction was performed in triplicate and results represent the mean of three replicates of one independent biological replicate. Data represent the mean relative expression and standard error obtained from one independent biological experiment. Different letters above the bars denote a significant difference (*P* ≤ 0.05, ANOVA) between samples by Tukey–Kramer multiple comparison test. The experiment was repeated twice for each housekeeping gene and similar results were obtained.

Among the standard criteria used for a secreted effector, genes associated with pathogenicity should be upregulated during nematode parasitism, indicating their regulation during disease development. For the expression-level assay during nematode development, we selected primers specific to *MjMCM2* (*M.Javanica*_Scaff2271g021798) to quantify its transcript level by qRT-PCR at five different stages of *M. javanica* development: eggs, ppJ2s, pJ2s 12 h after inoculation, J3–4s and female parasitic stages. We set the *MjMCM2* expression level in eggs to 1 in order to calculate the FC during *M. javanica*'s subsequent life stages. Highest expression of *MjMCM2* was found in the parasitic stages, where expression was increased by 2.3, 1.75 and 2.6 FC at the pJs, J3-4 and females stages respectively, compared with the egg stage (*p* ≤ *0.05*, ANOVA), indicating that MjMCM2 might play a role during parasitism (Fig. 2).

### *MjMCM2* transcripts are localized to ppJ2 esophageal glands

**Figure 3:**
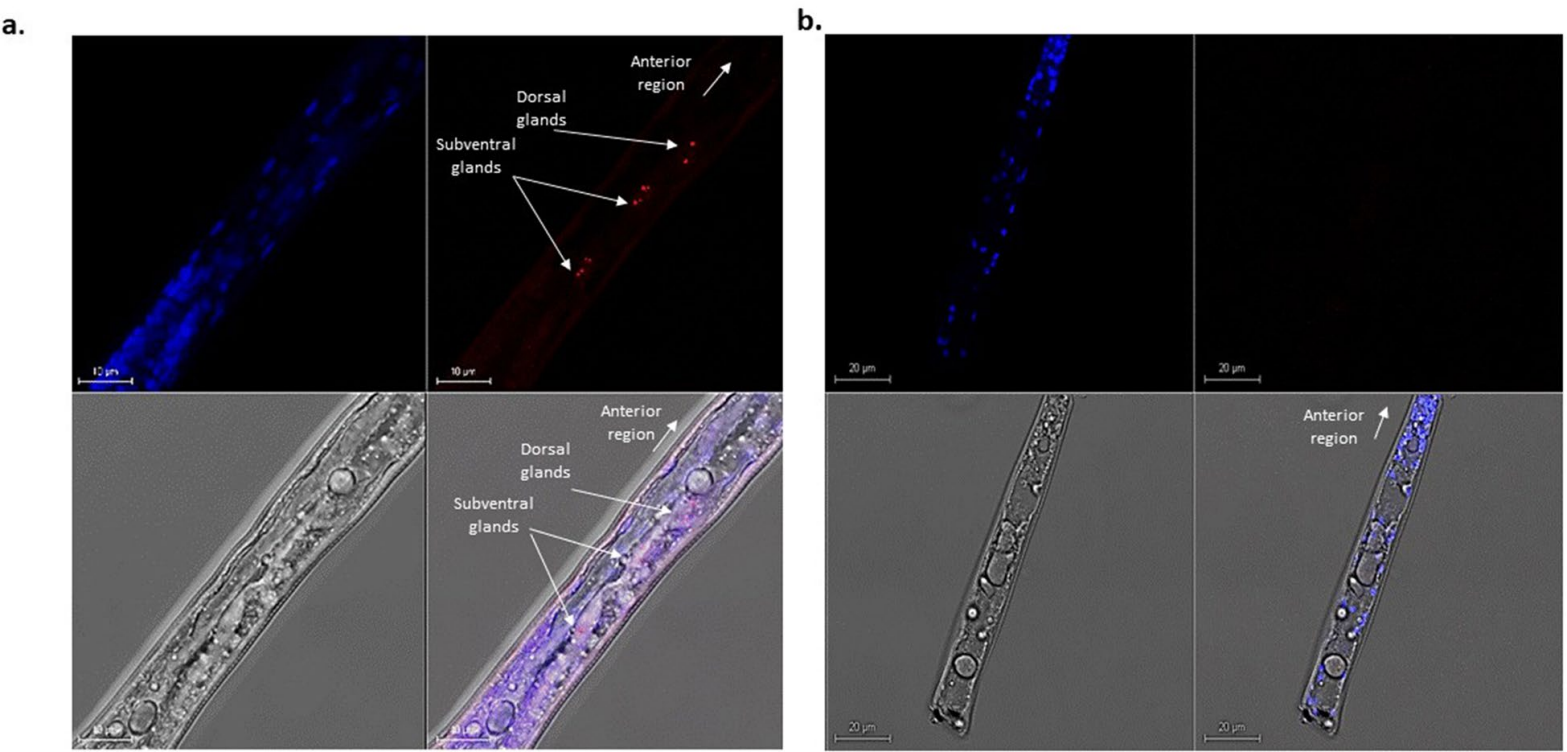
Localization of *MjMCM2* mRNA using a gene-specific *MjMCM2-*Cy5 cDNA-labeled probe (red) visualized in dissected *M. javanica* juvenile nematodes exposed to the oxylipin 9-HOT. *M. javanica* J2s were counterstained with DAPI (nuclei; blue). (**a**) J2s exposed to oxylipin 9-HOT and then subjected to FISH. *MjMCM2* mRNA fluorescence was visualized by fluorescence microscopy. Top: DAPI staining (left) and hybridization signal in red indicated by arrows (right). Bottom: *M. javanica* visualized by differential interference contrast (DIC) microscopy (left) and overlaid images with DAPI staining and hybridization signal (right). (**b**) Control J2s were subjected to FISH in 0.01 M MES buffer without exposure to 9-HOT, resulting in no hybridization signal. Samples were visualized under an IX81Olympus FluoView500 confocal microscope. Scale bar = 20 µm.

FISH was used to study the spatial location of *MjMCM2* in segmented freshly hatched *M. javanica* ppJ2s. We used a Cy5 probe on fixed ppJ2s following exposure to 9-HOT and as a control, those that were not exposed to the oxylipin. A strong signal was only observed in the esophageal gland of ppJ2s treated with 9-HOT and no signal was detected in ppJ2s that were untreated (Fig. 3b). *MjMCM2* expression in the nematode secretory gland strongly suggests its potential secretion as a nematode effector during parasitism (Fig. 3a).

### *In planta* subcellular localization indicates that MjMCM2 is targeted to the plasma membrane and the nucleus

**Figure 4:**
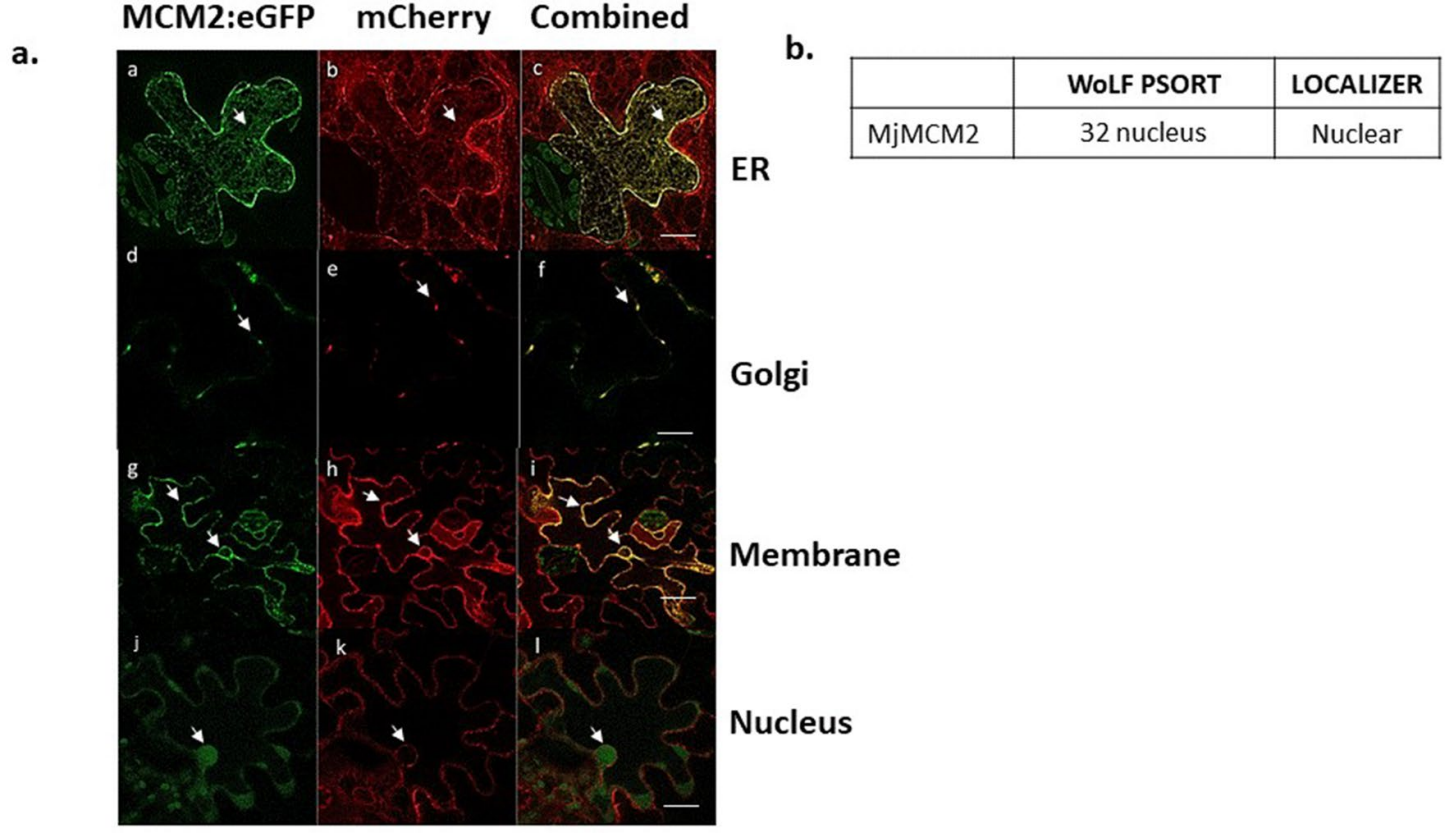
Subcellular localization of MjMCM2 tagged with eGFP in *N. benthamiana* leaves and apparent co-localization with endoplasmic reticulum, Golgi and plasma membrane. Organelle markers: mCherry–endoplasmic reticulum (ER-Rb; 35S::mCherry-HDEL), mCherry–Golgi (GmMan1-RFP) and mCherry–cytoplasmic membrane (aquaporin PIP2A:RFP). Samples were analyzed with HyD (hybrid) detectors ranging from 500 to 530 and 580 to 650 nm, respectively. Leaves were infiltrated with a 1:1 mixture of *A. tumefaciens* GV3010 containing (**a**–**c**) MjMCM2–eGFP (green) or ER-Rb (red); (**d**–**f**) MjMCM2–eGFP or Golgi-Rb(GmMan1-RFP) (red); (**g**–**i**) MjMCM2*–*eGFP or membrane-Rb(aquaporin PIP2A:RFP) (red); (**j**–**l**) MjMCM2*–*eGFP or membrane-Rb(aquaporin PIP2A:RFP) (red); (**a**,**d**,**g**,**j**) Images acquired with BA505–525 filter for GFP. (**b**,**e**,**h**,**k**) Images acquired with filter BA560IF for RFP. (**c**,**f**,**i**,**l**) Sequential acquisition and fusion of images acquired with both filters, overlap of GFP and RFP signals displayed in yellow. Scale bars: (**c**) 10 µm; (**f**) 5 µm; (**i**) 20 µm; (**l**) 10 µm. (**b**) Subcellular location results of MjMCM2 as predicted using WoLF PSORT (https://www.genscript.com/wolf-psort.html; and LOCALIZER (http://localizer.csiro.au/).

To determine the effector's target site once delivered into the plant cell, we expressed the *MjMCM2:eGFP* construct under the control of the CaMV-35S promoter in *N. benthamiana* leaf epidermal cells together with endoplasmic reticulum (ER-Rb; 35S::mCherry-HDEL), Golgi apparatus (GmMan1-RFP) and cytoplasmic membrane (aquaporin PIP2A:RFP) markers (Fig. 4). MjMCM2 co-localized with the endoplasmic reticulum and Golgi network, as seen by the yellow fluorescence (Fig. 4a–c,d–f, respectively). MjMCM2 signal was also observed in the plasma membrane, as well in the nucleus of some leaf epidermal cells (Fig. 4ag–i,j–l, respectively), as partly predicted by WoLF PSORT and LOCALIZER *in silico* (Fig. 4b). Free-eGFP control infiltrated leaves revealed a very faint and diffusive signal as observed in Fig. S4.

### MjMCM2 overexpression in tomato roots results in increased susceptibility upon *M. javanica* infection

**Figure 5:**
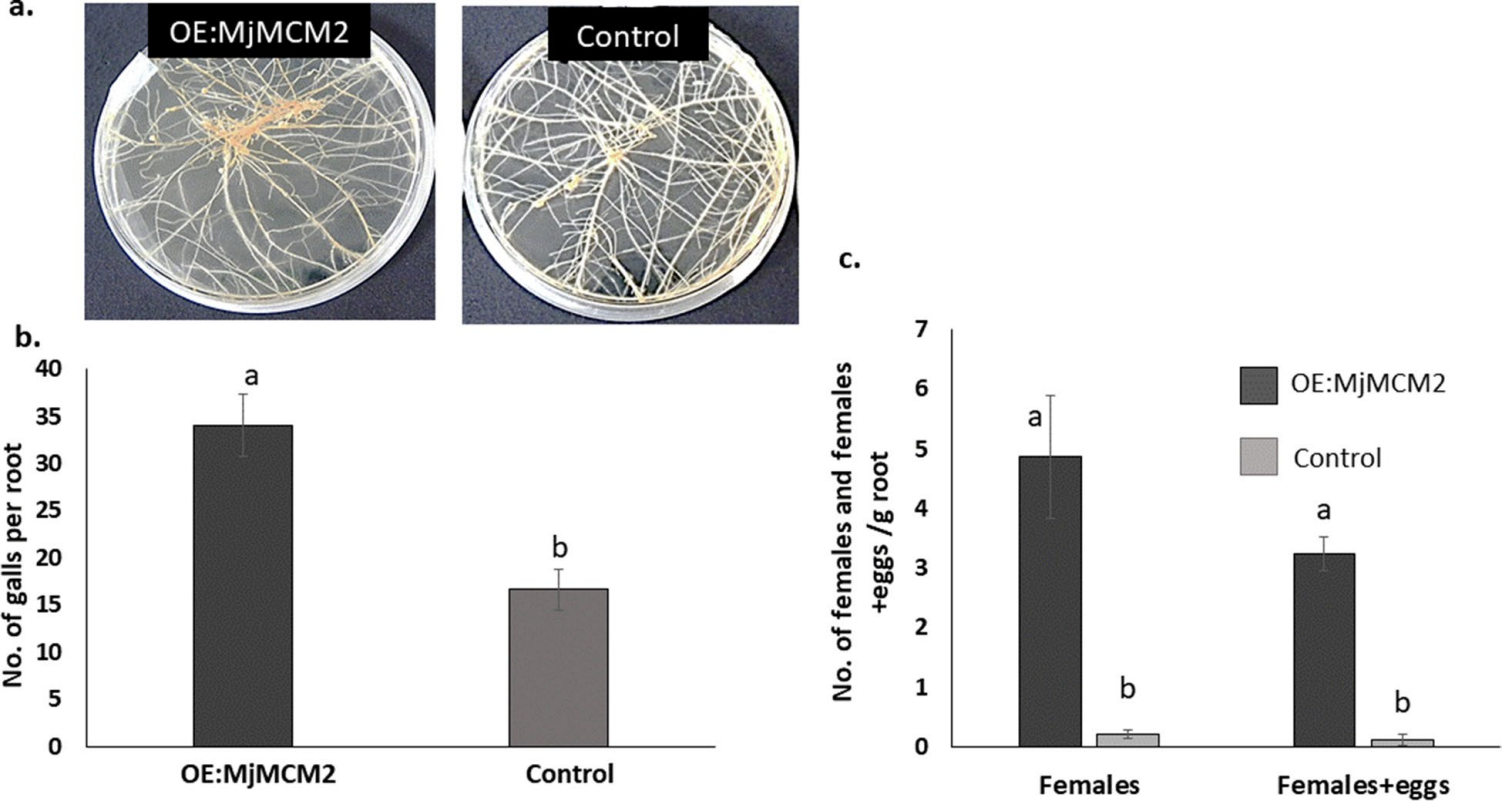
*MjMCM2-*overexpressing tomato roots are highly responsive to nematode infection. (**a**) Nematode-infected transgenic tomato hairy roots overexpressing *MjMCM2* (*MjMCM2*^*OE*^) and control roots at 28 DAI. (**b**) Mean number of galls counted on respective root systems at 28 DAI. (**c**) Means of dissected females and females + eggs per gram of tomato hairy root lines overexpressing *MjMCM2* (dark gray) compared to a control line in which roots were transformed with empty vector (light gray). Roots were inoculated with 200 *M. javanica* juveniles. Different letters above the bars denote a significant difference (*P* < 0.05, ANOVA) between the different tomato roots analyzed by Tukey–Kramer multiple comparison tests. Fifteen plates per root line were analyzed and experiment was repeated twice, generating similar results.

To evaluate the function of MjMCM2 during nematode infection, the construct pK7FWG2,0:MjMCM2 under the CaMV-35S promoter was expressed in tomato roots. The transgenic tomato hairy root lines overexpressing *MjMCM2* (*MjMCM2*^*OE*^) and control lines were inoculated with freshly hatched ppJ2s. At 28 DAI, galls were counted and roots were dissected to release and record the number of females and egg masses produced on the infected roots. Galls number produced on *MjMCM2*-overexpressing roots were increased by 100% compared with galls number measured on control roots (*P* ≤ 0.05, ANOVA; Fig. 5a,b). Moreover, more maturing and egg-laying females indicated a clear benefit for nematode development in *MjMCM2*-overexpressing roots compared to controls as observed by 5 and 3 fold increment, respectively (*P* ≤ 0.05, ANOVA; Fig. 5c). Root morphology, growth and branching were similar in *MjMCM2*^*OE*^ and control lines. Thus, infection and reproduction were promoted in transgenic roots overexpressing *MjMCM2*.

**Figure 6:**
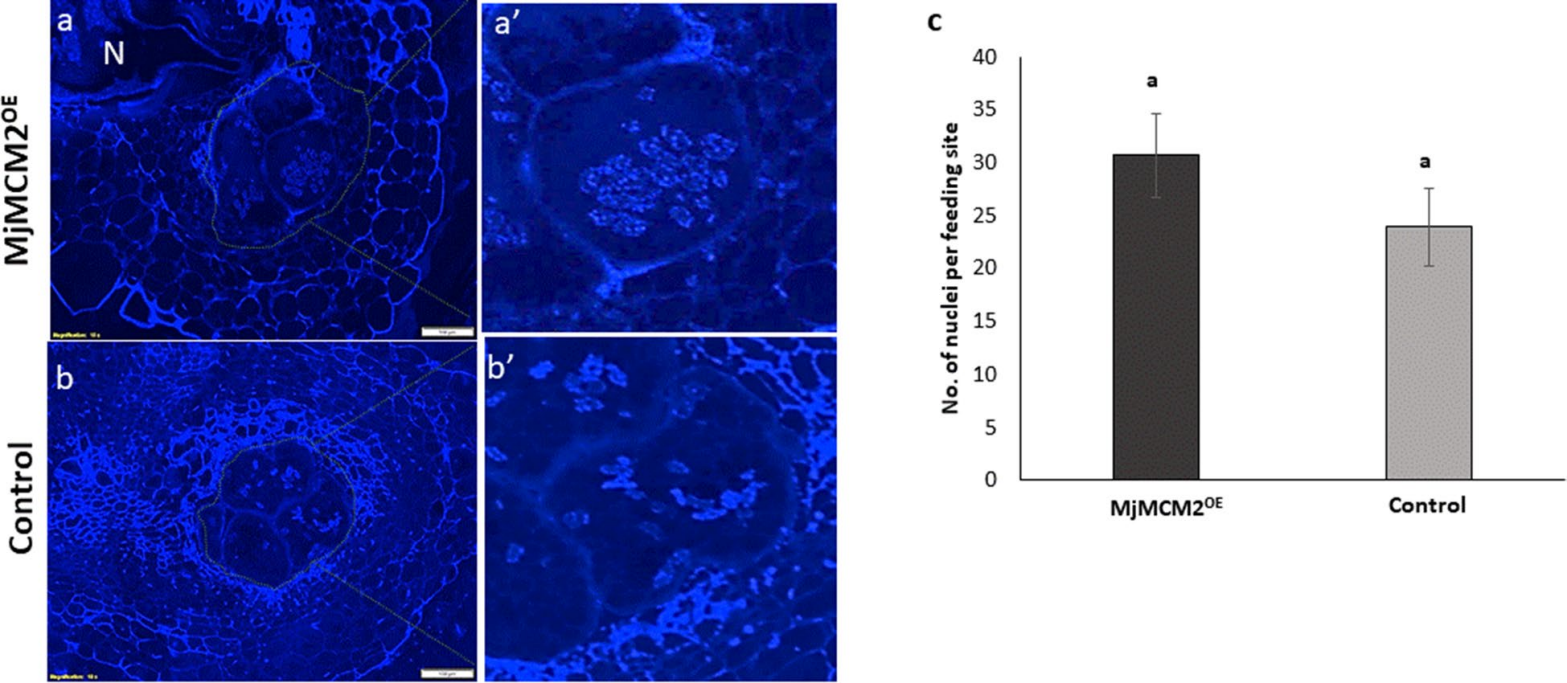
Cytological analyses of galls induced in *MjMCM2-*overexpressing (*MjMCM2*^*OE*^) lines compared to controls. Sections of galls in tomato hairy roots 28 d after *M. javanica* infection stained with DAPI. (**a**,**a’**) Gall in hairy roots overexpressing *MjMCM2* illustrating a higher density of nuclei. Feeding sites are encircled by dots and a giant cell (GC) is zoomed in on in (**a’**) showing apparently larger nuclei, with prominent chromocenters. (**b**,**b’**) Gall from control line (empty vector-transformed roots). Feeding sites are encircled by dots and a GC is zoomed in on in (**b’**). N, nematode. (**c**) Average number of nuclei per medium-sectioned feeding site of 15 GCs from 15 gall sections of *MjMCM2*^*OE*^ line(dark gray) compared to controls (light gray). Letters above the bars denote significant difference (*P* ≤ 0.05, ANOVA) between tomato root lines analyzed by Tukey–Kramer multiple comparison tests.

To further analyze the impact of *MjMCM2* on regulation of feeding-site development, we conducted morphological and cytological analyses of sectioned galls 28 DAI. Gall sections were stained with DAPI to visualize morphological changes in the feeding sites and distribution of nuclei (Fig. 6). Galls overexpressing *MjMCM2* showed apparently larger GCs (Fig. 6a,a’) that were more densely filled with larger nuclei harboring prominent chromocenters compared to the control (Fig. 6b,b’). More visible nuclei per section upon *MCM2* overexpression (Fig. 6c) suggests higher mitotic activity in these GCs.

### *In silico* protein-motif discovery of MjMCM2 reveals two RxLR-like motifs

**Figure 7:**
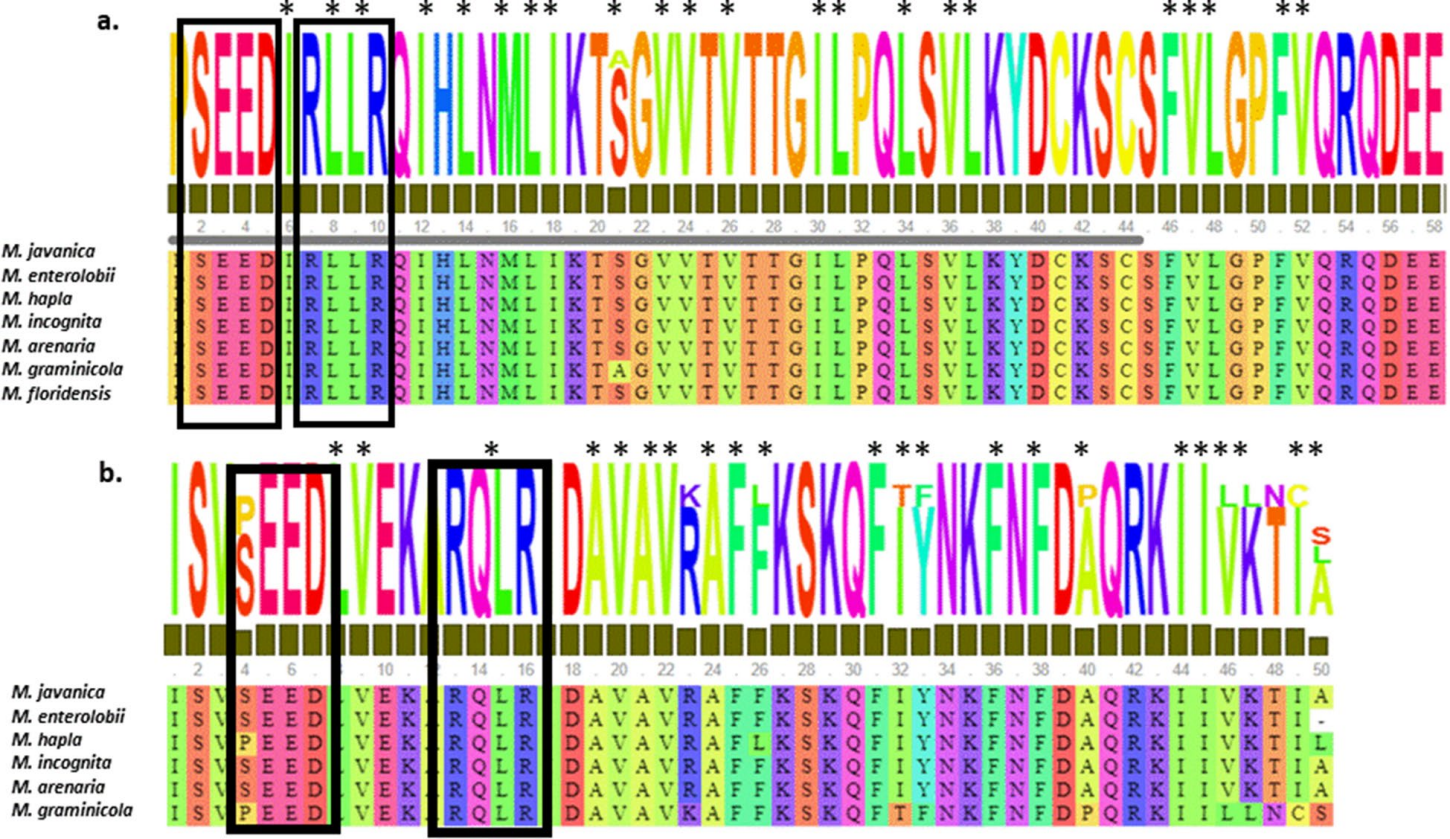
Occurrence of host-translocation RxLR-like motif in MjMCM2 proteins of seven *Meloidogyne* species. Alignment of (**a**) *M. incognita* (Minc3s02741g31411)*, M. javanica* (M.Javanica_Scaff2271g021798)*, M. hapla* (MhA1_Contig1624.frz3.gene11)*, M. arenaria* (M.Arenaria_Scaff6715g060087)*, M. enterolobii* (scaffold23895_cov224.g21986*), M. graminicola* (NXFT01004025.1.10522_g) and *M. floridensis,* which all possess the first RxLR motif (in black rectangle) close to the N-terminal preceded by the SEED motif (in black rectangle). (**b**) Second RxLR motif (in black rectangle) sequences were found in six *Meloidogyne* species (*M. floridensis* is lacking the C-terminal sequence)*.* The second RxLR2 was preceded by a SEED motif in most of the RKN species; exceptions were *M. hapla* and *M. graminicola*, where it was preceded by a PEED-motif. Asterisks mark hydrophobic amino acids, indicated by WYL or WY motifs.

To further investigate the potential secretion of MjMCM2 (*M. Javanica_*Scaff2271g021798), we searched for regulatory sequence motifs and conserved secretion motifs using Motif Scan (http://myhits.isb-sib.ch/cgi-bin/motif_scan/). Among the various known motifs, a conserved N-terminal Arg–Xaa–Leu–Arg (RxLR) translocation motif, which is common in oomycete cytoplasmic effectors, appeared twice along the protein sequence (Supporting Information Fig. S1, Fig. 7).

In general, the RxLR motif is defined by the sequence Arg–x–Leu–Arg, where x is any amino acid, and in some cases it is followed by an acid-rich DEER motif (Asp–Glu–Glu–Arg). Sequence analysis of *MjMCM2* indicated that it does not contain a signal peptide for secretion, or any transmembrane domain. However, further investigation revealed the presence of an acidic region characterized by a SEED (Ser–Glu–Glu–Asp) motif followed by an RxLR-like region positioned at M-162-SEED-1-RLLR-3-L-1-KYK-2-FV-4-L-K-2-F-V-L (Fig. 7a) and at M-753-SEED-1-RQLR-3-V-2-FKFYF (Fig. 7b). The presence of MCM2 sequences was also investigated in other *Meloidogyne* spp., and in other available *Meloidogyne* spp. genomes: *M. enterolobii* (seq number), *M. hapla* (MhA1_Contig1624.frz3.gene11), *M. incognita* (Minc3s02741g31411), *M. arenaria* (*M.Arenaria*_Scaff6715g060087), *M. graminicola* (NXFT01004025.1.10522_g), *M. floridensis* (M. *floridensis* 1.1.scaf04253), and these were aligned using ClustalW program^55^. Sequence alignments indicate a conserved SEED–RxLR-like motif in nematodes belonging to the *Meloidogyne* family (Fig. 7).

**Figure 8:**
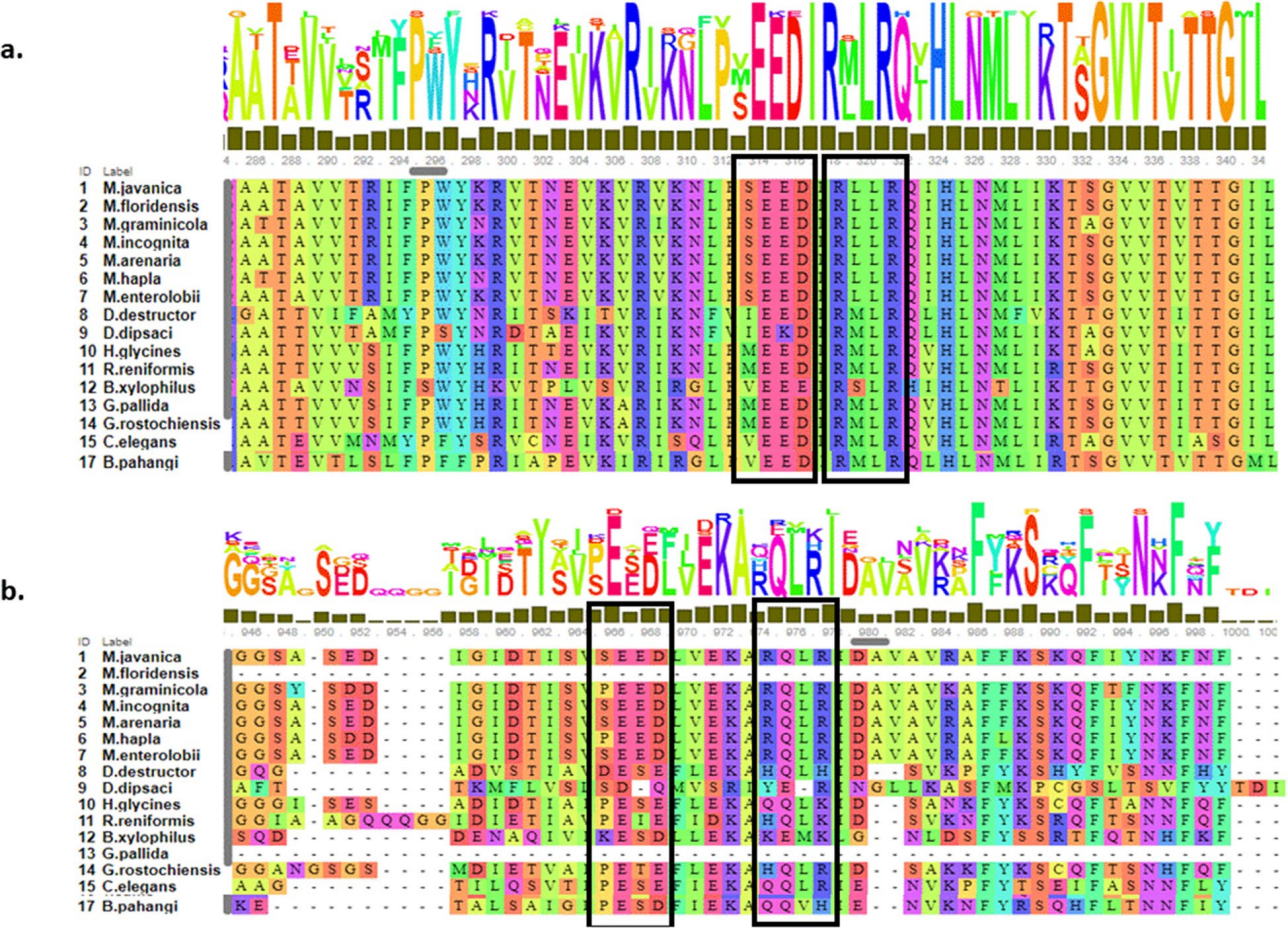
Occurrence of host-translocation RxLR-like motif in MCM2 proteins of 16 species of nematodes. (**a**,**b**) Alignment of all seven *Meloidogyne* spp. shown in Fig. 7 with cyst nematodes *Globodera pallida* (GPLIN_000686900), *Globodera rostochiensis* (GROS_g09797.t1), *Heterodera glycines* (Hetgly.G000003842); migratory endoparasites *Bursaphelenchus xylophilus* (BXY_0765600), *Ditylenchus destructor* (Dd_02935), *Ditylenchus dipsaci* (jg5302); semi-endoparasitic nematode *Rotylenchus reniformis* (TRINITY_GG_28486_c36_g1_i1); free-living, bacterial feeder nematode *Caenorhabditis elegans* (BX284602.5), and animal nematode *Brugia pahangi* (Bm6301b.1). (**a**) First RxLR1-like following the SEED motif region (in black rectangle) and (**b**) second RxLR-like motif following the SEED motif region (in black rectangle).

This motif was also present in available MCM2 sequences for other PPNs belonging to the suborder *Tylenchida* such as the cyst nematodes *Globodera pallida* (GPLIN_000686900), *Globodera rostochiensis* (GROS_g09797.t1) and *Heterodera glycines* (Hetgly.G000003842), the migratory endoparasites *Bursaphelenchus xylophilus* (BXY_0765600), *Ditylenchus destructor* (Dd_02935) and *Ditylenchus dipsaci* (jg5302), the semi-endoparasitic nematode *Rotylenchus reniformis* (TRINITY_GG_28486_c36_g1_i1), and members of suborder *Rhabditida*, i.e., the free-living bacterial feeder nematode *Caenorhabditis elegans* (BX284602.5), and the animal nematode *Brugia pahangi* (Bm6301b.1). Thus, whereas the first RxLR motif was common among all nematodes, the second SEED–RxLR-like motif was solely found in *Meloidogyne* spp. (Fig. 8).

### MjMCM2 3D homology modeling

Homology modeling is a standard structure-prediction method that contributes to understanding the relationship between protein structure and function. To investigate the molecular bases of the different functions elicited by the second RxLR, the 3D structure of MjMCM2 was predicted by homology modeling, using the SWISS-MODEL server^51^. The Local Quality plot (https://swissmodel.expasy.org/docs/help#qmean) showed that the predicted structure contains mainly two large regions of good local alignment (QMEANDisCo > 0.60), where the second region contains the second RxLR (Supporting Information Fig. S2, inset shows sequence of residues surrounding the second RxLR). Secondary-structure representation demonstrated that the second RxLR is positioned on an exposed loop (Supporting Information Fig. S3a). Surface representation of the molecular model showed that the RLR residues of the second RxLR motif were on the surface in a polar environment (Supporting Information Fig. S3b, blue indicates polar), inside the region with the high local alignment score (QMEANDisCo) relative to the template used for the homology modeling (Supporting Information Fig. S3a).

### *M. javanica* genome mining for sequences encoding proteins carrying a RxLR-like motif

To further study the occurrence of RxLR-like motifs in the *M. javanica* genome, we conducted a comprehensive *de novo* gene prediction of RxLR-like motifs on *M. javanica* ORFs using the *effectR* program developed by Tabima and Grünwald^48^ as a genome mining tool. This program enables rapid and reproducible prediction of effectors in oomycete genomes, or with custom scripts in any genome. The *effectR *package relies on a combination of regular expression statements and hidden Markov model approaches to predict candidate RxLR motifs ^48^.

For the genome search, three scripts were used (Supporting Information Table S1): a strict motif pattern—the conserved RxLR–DEER motif (*RxLR), resulting in the identification of 217 candidate proteins, of which only two proteins carried a canonical signal peptide; the motif relax mode—**RxLR, resulting in 1305 candidate proteins, of which only 12 proteins carried a canonical signal peptide; and the motif relax2 mode—***RxLR, resulting in 2525 candidate proteins, of which 54 carried a canonical signal peptide. A conserved and complete RxLR–DEER-like motif was found on two different scaffolds encoding superoxide dismutase (SOD), and one encoding α-amylase (Supporting Information Table S2). Degenerate RxLR motif was also found in cell wall-degrading enzymes, such as the arabinanase gene required for polysaccharide degradation^56^, and the glycoside hydrolase (GH) that catalyzes the hydrolysis of the glycosidic linkage of glycosides^57^ (Supporting Information Table S2).

## Discussion

The establishment of galls induced by RKNs involves a number of alterations in the plant host root that are essentially driven by secreted molecules. These molecules likely cause the dedifferentiation of vascular cells into GCs, forming a feeding site upon which the nematode depends to lay eggs that will hatch into new infective juveniles. These GCs are highly dependent on cell-cycle activity leading to multinucleation via acytokinetic mitosis, followed by nucleus enlargement caused by the action of the endocycle. Stimulation of the host's cell-cycle machinery may be potentially controlled by candidate secreted effectors, as part of the nematode's manipulation of host gene expression, but these and their targets remain to be identified^11,58^. For DNA synthesis during the S phase, genes involved in mitosis as well as the endocycle, such as *ORC1-6*, *MCM5, CDT1a,b* and *CDC6*, have been found to be expressed in GCs, characterized by the prominent DNA synthesis needed for their cell-cycle machinery^8^. Several potentially secreted nematode proteins—a CDC48-like protein, a ubiquitin, and a SKP1-like protein—with possible roles in cell-cycle regulation have been identified *in silico*^59^. However, so far, their role in cell-cycle regulation during feeding-site formation and development has not been demonstrated.

Herein, we uncover the first candidate effector gene, *MCM2* of *M. javanica*, which is likely secreted and may directly or indirectly affect the host cell cycle. MjMCM2 might act by mimicking the function of plant-endogenous MCM2 as a licensing factor, facilitating the transformation process of root cells into large multinucleate feeding cells.

### The *M. javanica* genome has multiple copies of *MCM2* genes

*M. javanica* reproduce by mitotic parthenogenesis, as they have a polyploid genome with highly divergent genome copies, apparently resulting from hybridization events, ploidy changes and chromosomal fragmentation^60^. Homologous gene copies generally exhibit different gene-expression patterns^61^. *M. javanica* has 26,917 predicted genes and 944 pseudogenes (BioProject PRJNA340324), despite the latter’s exhibit general homology to known genes, they are not functional. In addition, transposable elements occupy ~ 50% of the genome, providing genome plasticity^60^. These previous observations might explain the various *MCM2* gene copies found in *M. javanica*. *MjMCM2* (*M.Javanica*_Scaff2271g021798) lacked a canonical signal peptide and transmembrane helices. As previously reported, many secreted effectors have been found to lack a predicted signal peptide^62,63^. In addition, *in silico* approaches have limitations: accurate N-terminal annotation is critical for signal peptide identification, signal peptides are highly heterogeneous, and some of them are indeed difficult to predict^64,65^.

### *MjMCM2* is expressed in the nematode esophageal gland and is upregulated during parasitic stages

To determine whether *MjMCM2* expression plays a role during nematode parasitism, we assessed its organellar location and its expression during the nematode's parasitic stages. FISH clearly localized MjMCM2 transcripts to the ppJ2 esophageal gland, the main secretion organ of PPNs. This strongly supports the notion that MjMCM2 is a secreted protein. Furthermore, qRT-PCR strongly suggested that *MjMCM2* expression is not only predominantly induced upon nematode penetration into the plant root, but is also continuously expressed during feeding-site establishment, supporting that MCM2 might be positively affecting the repeated mitosis cycles occurred in the nematode feeding sites.

### MjMCM2 co-localizes with the plasma membrane, endoplasmic reticulum and nucleus of *N. benthamiana* leaves

Localization of MjMCM2 in *N. bethamiana* leaves revealed its potential function in the plant host during nematode infection. Thus, an assay was performed to determine the cellular compartments that this protein might target when secreted in GCs during parasitism. Subcellular localization of our potentially secreted effector MjMCM2 fused to eGFP in *N. benthamiana* leaves was tracked along the plasma membrane and in the nucleus as predicted *in silico*. MjMCM2 localization in the endoplasmic reticulum and Golgi apparatus suggested that this protein might enter the cell's secretory pathway, and indicated its recognition by the plant machinery as described previously by Jaouannet et al.^66^, when studying the Mi-CRT effector localization. In addition, we must consider that some effectors must undergo modifications to be targeted to their subcellular localization, as well as for their biological activity^67,68^. Thus post-translational modifications might occur after MjMCM2 secretion so that it can properly perform its function, as a complex that is imported into the nucleus to be assembled into a pre-replication complex at the M/G1 cell-cycle stage^69,70^. Moreover, nematode effectors that are targeted to the plant nucleus are predicted to be involved in the regulation of the plant cell cycle. Since GCs are also connected to neighboring cells by plasmodesmata^71^, it could be speculated that secreted proteins such as MCM2 might diffuse to, and be involved in cell-cycle activation in other gall cells in the vascular tissue where the gall is located.

Moreover, the change observed in nuclei appearance in the overexpressing galls might be the result of increased mitotic cycles. In addition, enlarged nuclei harboring multiple large chromocenters suggests increased endoreduplication, likely facilitated by MjMCM2 overexpression. Changed nucleus morphology in GCs is consistently seen during functional studies of cell-cycle genes ^12–15,72^. Furthermore, Kondorosi and Kondorosi^73^, have shown that in *Arabidopsis* the endocycle induce key S-phase genes such as *ORC*, *CDC6, CDT1* and *MCM* genes. Interestingly most of these genes are somewhat expressed in galls^74^, supporting their implication in typical endocycle route during nematode feeding site establishment and maintenance. Findings observed here, are also in a good agreement with previous studies demonstrated the early upregulation of key components of the core cell cycle machinery in nematode feeding sites (CDK,1, CDKB1,1, CYCA2,1 and CYCB1;1 as shown by promoter activity and transcript localization^3,11^. Given that MCM2-7 is targeted by several different kinases including CK2, cyclin-dependent kinases (CDK)^75^, their co-occurrence in nematode feeding sites might ensure its modulation.

The process of identifying genes encoding candidate effectors in the nematode genome has become an important tool, as these proteins are directly involved in pathogenicity.

We know that some effectors are secreted through non-classical secretion pathways. We also know that RKNs secrete some effectors from their stylets into the apoplast^76^, and yet data suggest that these effectors are translocated to, and function inside the plant cells. There is a need to study RKNs for non-canonical secretion and effector translocation into host cells. This led us to look at other pathogens and their effectors. For example, there are oomycete effectors containing a signature RxLR motif^77^. This motif is implicated in translocation of the pathogen proteins into host cells^78,79^. Interestingly, signal peptide-lacking RxLR effector proteins have been shown to be secreted unconventionally^63,80^. In the past, there has been limited genetic information for PPNs, and initial scans of PPN genomes concluded that there were no effectors with RxLR or similar translocation motifs^81,82^. However, more recent work has identified RxLR motif-containing genes in the cyst nematode *Heterodera avenae*. In a transcriptome analysis comparing preparasitic to parasitic *H. avenae*, 61 transcripts were identified that encoded proteins predicted to have secretory peptides and an RxLR motif. These proteins were identified as putative cyst nematode RxLR effectors^83^. Herein we provide the first evidence of an effector with conserved RxLR motifs being present and functioning in PPNs.

Our observation of two RxLR-like motifs along the amino acid sequence of MjMCM2 raises substantial questions about its function in translocation to the host plant. One such question is whether the two motifs found in MjMCM2 potentially exert similar effector translocation behavior as the single RxLR-motif in oomycetes^49,84,85^. The RxLR motif was originally identified by comparing sequences of effectors from *Hyaloperonospora arabidopsidis, Phytophthora infestans* and *Phytophthora sojae*^86^. Following intensive studies, the RxLR-motif is now considered important for translocation of oomycete effectors into plant cells^79,87^. Localization of *MjMCM2* to the nematode secretory glands and its overexpression *in planta* strongly suggest its secretion into the host root.

While no RxLR effector proteins have been found in RKNs^88^, herein we suggest the occurrence of a degenerate RxLR-like motif displayed as a SEED––RxLR motif twice in the MjMCM2 protein. Our *in silico* analysis encompassing several nematode species demonstrated the first RxLR-like motif in all available MCM2 amino acid sequences. However, the second RxLR-like motif observed here was solely found in *Meloidogyne* spp., it was absent in other Tylenchida PPNs and in other free-living Rhabditida nematodes*.* Molecular homology modeling predicted the position of the second RxLR-like domain on a surface-residing polar loop. As surface loops have been suggested to participate in protein–protein interactions^89^, this result supports its postulated interactive function with other proteins. Similarly, two adjacent RxLR motifs were found in the effector protein Avr1b from *Phytophthora sojae*, although the RxLR-motif was found not to be essential for avirulence function^79^. Future studies should focus on the functional aspects of these RxLR-like motifs, particularly through direct mutagenesis. Further mining of the *M. javanica* genome should be performed using the *effectR* package, designed to predict effector proteins, including other RxLR effector proteins. Use of this approach by Tabima and Grünwald^48^ resulted in the discovery of several genes in the *M. javanica* genome, e.g., SOD and α-amylase, whose secretion and function have been studied during parasitism by other PPNs^90,91^. Similarly, other cell wall-associated proteins, such as GH which catalyzes the hydrolysis of the glycosidic linkage of glycosides^57^, the 5-L-arabinanase 1 gene that is required for polysaccharide degradation^56^ and a laminin-like protein that interacts with receptors anchored in the plasma membrane of cells adjacent to basement membranes^92^, have all been shown to carry degenerate RxLR-like motifs.

## Concluding remarks

Overall, our study places MjMCM2 as the first candidate effector that might directly affect the mitotic and endoreduplication cycles in RKN-induced GCs, during gall genesis. Our localization studies strongly suggest that this protein is secreted by the nematode and that it can potentially be transported to the nucleus after possible structural modifications in the endoplasmic reticulum and Golgi bodies. Overexpression data suggest the importance of this MjMCM2 for parasitism, suggesting the use of these data for application in crop species, for e.g., by expressing double-stranded MjMCM2 *in planta*, then silencing it in the nematode. The discovery of two RxLR-motifs in this MjMCM2 protein has to be further investigated to determine whether MjMCM2 translocation into the plant follows a non-conventional pathway. Furthermore, it would be interesting determine the relevance and function of this repeated motif. These findings might contribute to distinguishing RKNs as that have the ability to induce multinucleate feeding cells and help us understand the molecular mechanisms governing plant–nematode interactions.

## Supplementary Information

Supplementary Figure S1.

Supplementary Figure S2.

Supplementary Figure S3.

Supplementary Figure S4.

Supplementary Table S1.

Supplementary Table S2.

## Data Availability

The data that support the findings of this study are available from the corresponding author upon reasonable request.
