# Supplementary figures and images for: The Minichromosome Maintenance Complex Component 2 (MjMCM2) of Meloidogyne javanica is a potential effector regulating the cell cycle in nematode-induced galls
Source: Sci Rep. 2022 Jun 2;12:9196. doi: 10.1038/s41598-022-13020-8 (PMC9163083; doi:10.1038/s41598-022-13020-8)

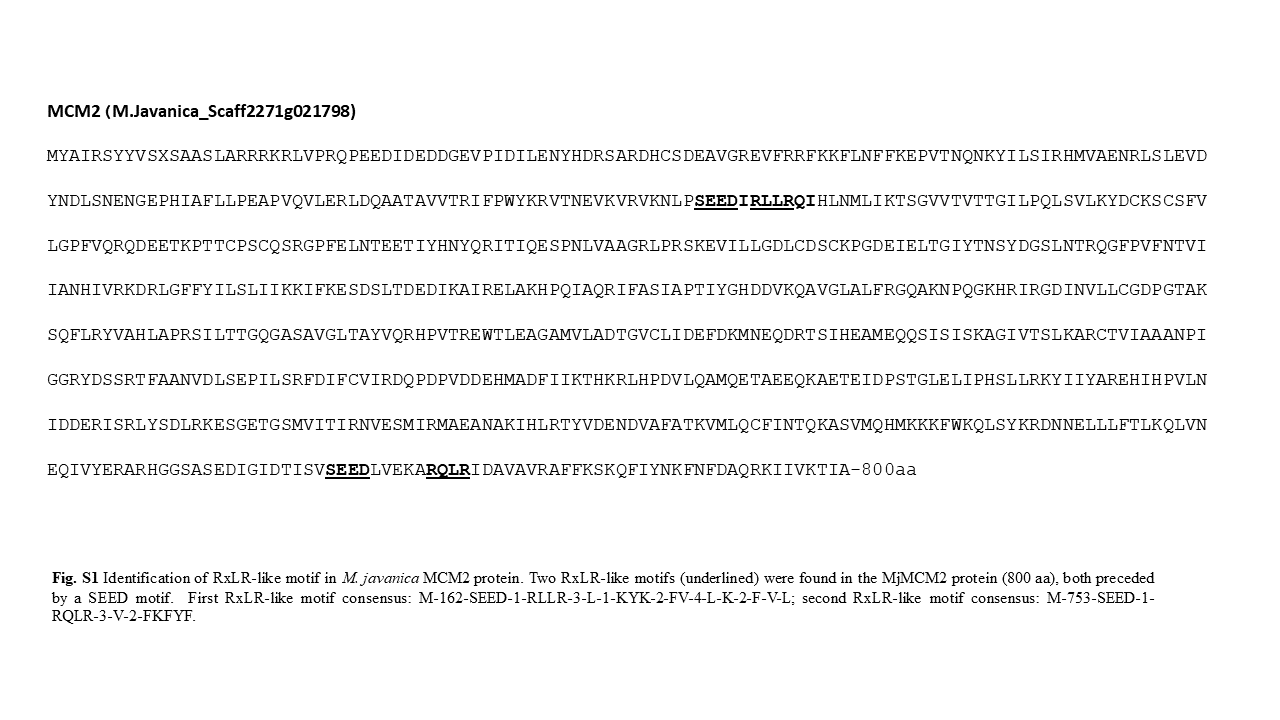

Supplement: Supplementary file 1 — Supplementary Figure S1. [file 41598_2022_13020_MOESM1_ESM.gif]

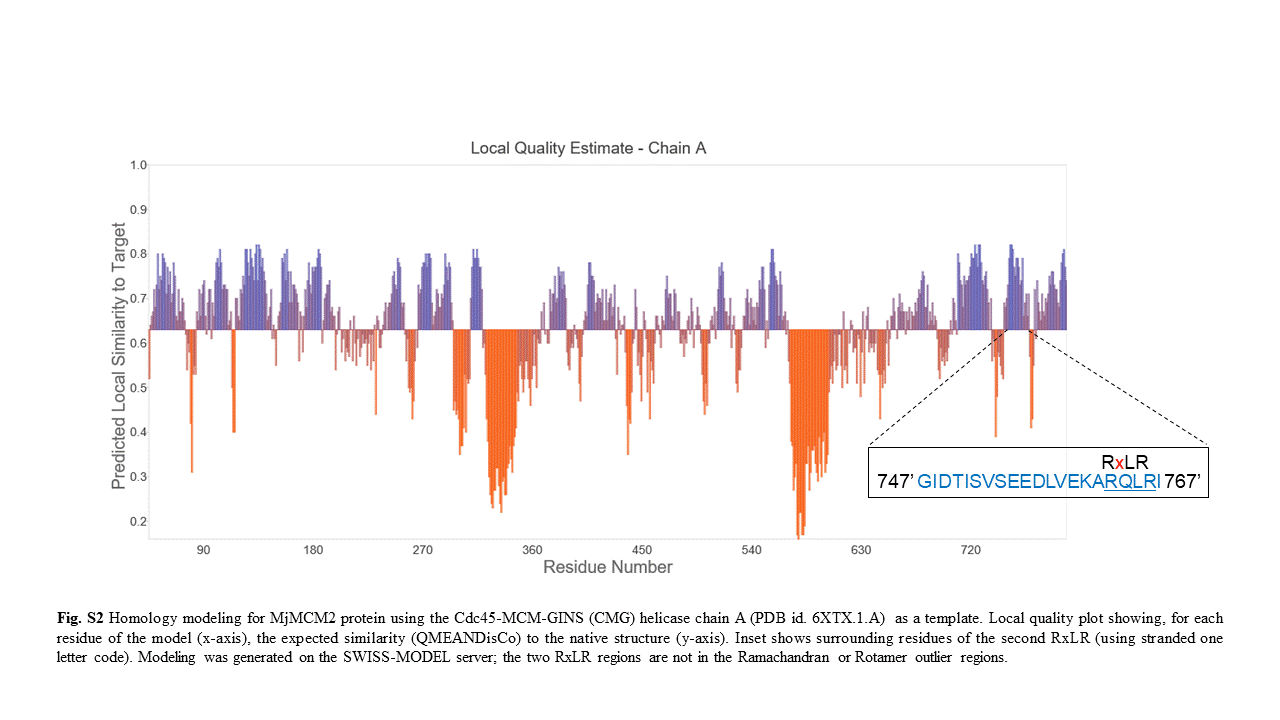

Supplement: Supplementary file 2 — Supplementary Figure S2. [file 41598_2022_13020_MOESM2_ESM.gif]

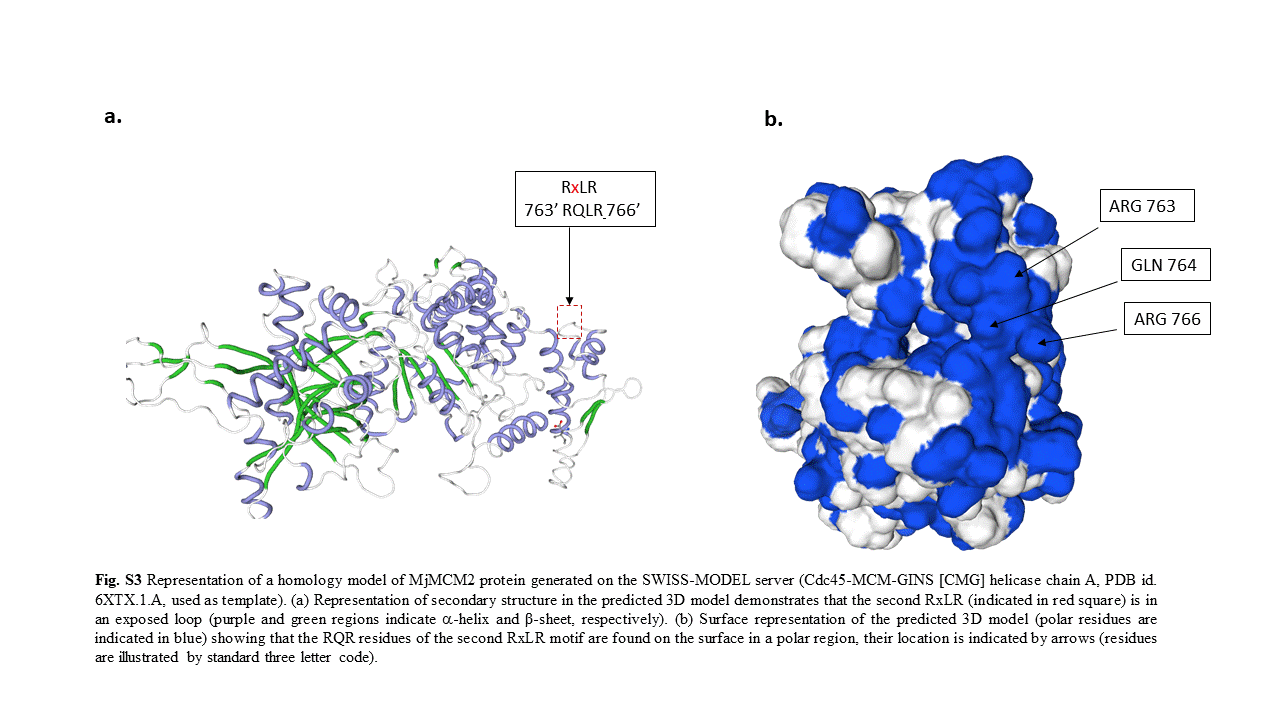

Supplement: Supplementary file 3 — Supplementary Figure S3. [file 41598_2022_13020_MOESM3_ESM.gif]

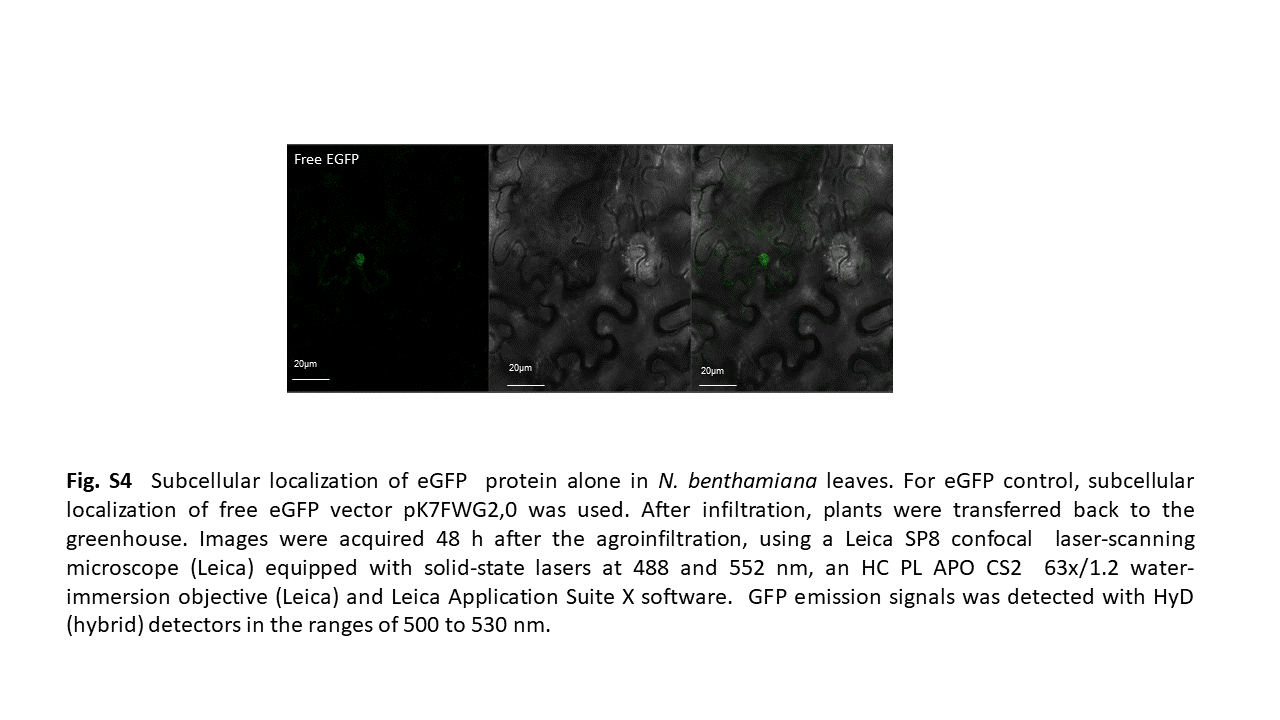

Supplement: Supplementary file 4 — Supplementary Figure S4. [file 41598_2022_13020_MOESM4_ESM.gif]
