# Supplementary material for: The Minichromosome Maintenance Complex Component 2 (MjMCM2) of Meloidogyne javanica is a potential effector regulating the cell cycle in nematode-induced galls
Source: Sci Rep. 2022 Jun 2;12:9196. doi: 10.1038/s41598-022-13020-8 (PMC9163083; doi:10.1038/s41598-022-13020-8)
Supplement: Supplementary file 5 — Supplementary Table S1. [file 41598_2022_13020_MOESM5_ESM.pptx]

## Slide 1
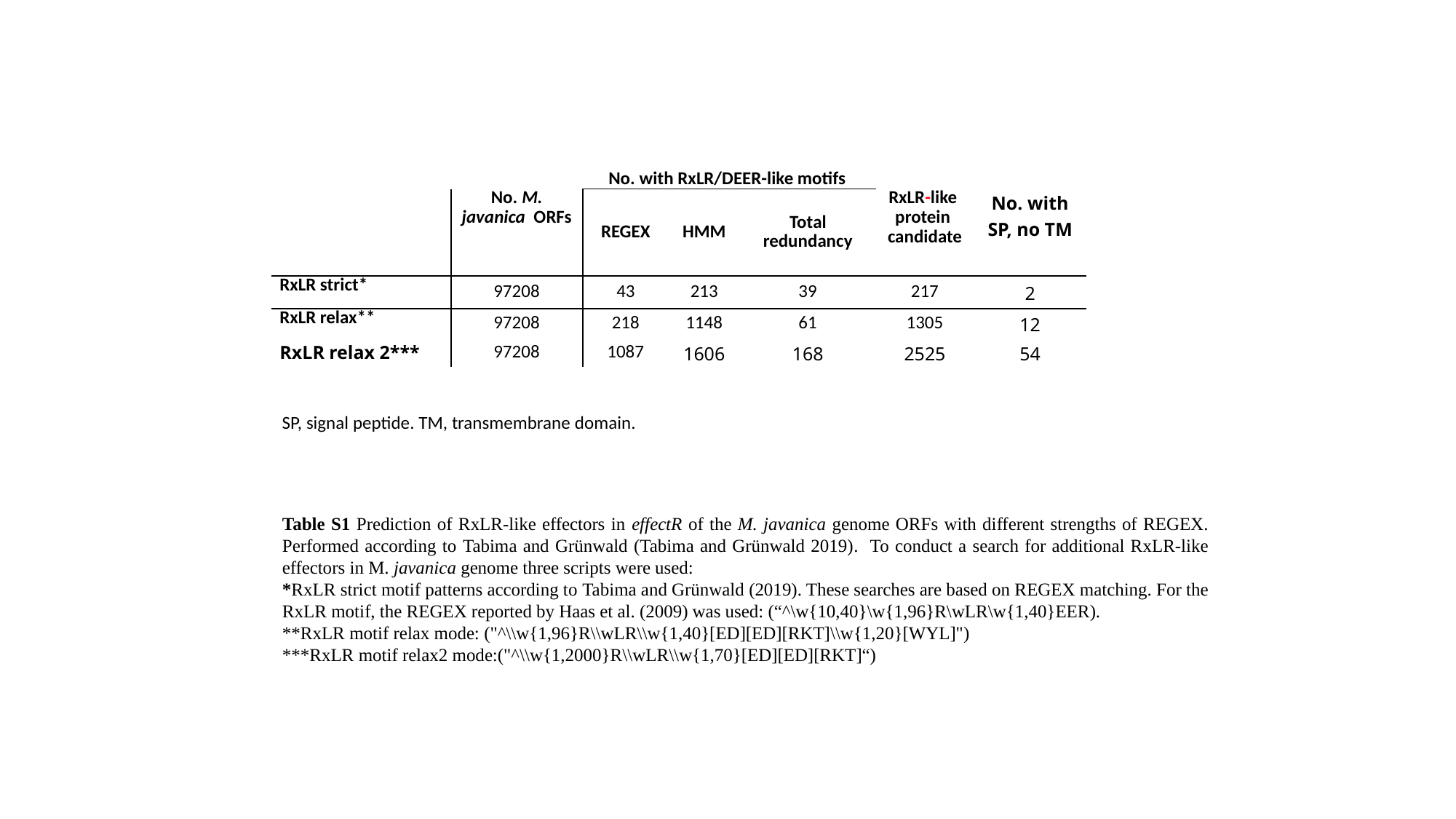

| | | No. with RxLR/DEER-like motifs | | | | |
| --- | --- | --- | --- | --- | --- | --- |
| | No. M. javanica ORFs | REGEX | HMM | Total redundancy | RxLR-like protein candidate | No. with SP, no TM |
| RxLR strict\* | 97208 | 43 | 213 | 39 | 217 | 2 |
| RxLR relax\*\* | 97208 | 218 | 1148 | 61 | 1305 | 12 |
| RxLR relax 2\*\*\* | 97208 | 1087 | 1606 | 168 | 2525 | 54 |
SP, signal peptide. TM, transmembrane domain.
Table S1 Prediction of RxLR-like effectors in effectR of the M. javanica genome ORFs with different strengths of REGEX. Performed according to Tabima and Grünwald (Tabima and Grünwald 2019). To conduct a search for additional RxLR-like effectors in M. javanica genome three scripts were used:
*RxLR strict motif patterns according to Tabima and Grünwald (2019). These searches are based on REGEX matching. For the RxLR motif, the REGEX reported by Haas et al. (2009) was used: (“^\w{10,40}\w{1,96}R\wLR\w{1,40}EER).
**RxLR motif relax mode: ("^\\w{1,96}R\\wLR\\w{1,40}[ED][ED][RKT]\\w{1,20}[WYL]")
***RxLR motif relax2 mode:("^\\w{1,2000}R\\wLR\\w{1,70}[ED][ED][RKT]“)
