# Supplementary material for: The Minichromosome Maintenance Complex Component 2 (MjMCM2) of Meloidogyne javanica is a potential effector regulating the cell cycle in nematode-induced galls
Source: Sci Rep. 2022 Jun 2;12:9196. doi: 10.1038/s41598-022-13020-8 (PMC9163083; doi:10.1038/s41598-022-13020-8)
Supplement: Supplementary file 6 — Supplementary Table S2. [file 41598_2022_13020_MOESM6_ESM.pdf]

Table S2 List of selected candidate RxLR effectors derived from *effectR* and carrying a degenerated RxLR-like motif. Secretion likelihood was calculated for each protein using the SignalP 5.0 server and SecretomeP 2.0 server.

Seq Name	Description	Length	RxLR Start	RxLR Motif	SignalP
**Coping with oxidative stress proteins**					
**M. javanica_Scaff24603g089596**	KJH49643.1copper/zinc superoxide dismutase	368	104	RELR	Sp 0.0022<br>SecP 0.561
**M.Javanica_Scaff436g006256**	zinc superoxide dismutase	567	124	RELR	Sp 0.9951<br>SecP 0.158
**Cell-cycle regulator**					
**M. javanica_Scaff12914g066007**	VIO95430.1 CDC5 protein, putative	655	192	RELR	Sp 0.0003<br>SecP 0.307
**M.Javanica_Scaff134g002415**	XP_001901183.1cell cycle-regulated factor p78	525	299	RELR	Sp 0.0004<br>SecP 0.405
**M.Javanica_Scaff2271g021798**	VIO94497.1DNA replication licensing factor MCM2, putative	800	168<br>763	RLLR<br>RQLR	Sp 0.0017<br>SecP 0.068
**M. javanica_Scaff3561g029820**	XP_018299661.1 cyclin-related protein FAM58A isoform X1	229	114<br>133	RCLR<br>RLLR	Sp 0.0077<br>SecP 0.078
**Cell wall associated**					
**M.Javanica_Scaff5917g041634**	CDJ83353.1Glycoside hydrolase domain containing protein	553	478	RSLR	Sp 0.967<br>SecP 0.148
**M.Javanica_Scaff549g007494**	5-L-arabinanase 1	131	43	RSLR	SecP 0.545<br>Sp 0.054
**M. javanica_Scaff489g006850**	KHN72422.1Laminin-like protein epi-1	4453	2328	RGLR	Sp 0.9731<br>SecP 0.796
**Cell metabolism and nutrition**					
**M. javanica_Scaff22595g086427**	XP_001899998.1Alpha amylase, catalytic domain containing protein	232	95	RSLR	Sp 0.3822<br>SecP 0.08
**M.Javanica_Scaff563g007635**	KHN76296.1ER degradation-enhancing alpha-mannosidase-like protein 1	581	505	RSLR	Sp 0.967<br>SecP 0.160
**M.Javanica_Scaff2667g024454**	KHN84842.1Zinc metalloproteinase dpy-31	315	56	RRLR	Sp0.9651<br>SecP 0.056
**M.Javanica_Scaff1607g017013**	XP_024501469.1Protease-associated domain	381	291	RRLR	SP 0.378<br>SecP 0.276
**M. javanica_Scaff22913g086941**	XP_024507885.1Glutaryl-Coenzyme A dehydrogenase	269	10	RTLR	Sp 0.0038<br>Secp 0.142
**M. javanica_Scaff7372g047821**	XP_020306027.1vacuolar protein sorting-associated protein 54	127	104	RALR	Sp 0.0005<br>SecP 0.959
**M. javanica_Scaff31269g098493**	GBM86013.1Glucose dehydrogenase [FAD, quinone]	278	109	RSLR	Signal P 0.0038<br>SecP
**M. javanica_Scaff7527g048450**	CEF62898.1 Sulfurtransferase	159	119	RLLR	Signal P 0.019<br>SecP 0.4622
**M. javanica_Scaff219g003587**	Proline dehydrogenase	661	136	RILR	SP 0.001<br>Secp 0.129
**M. javanica_Scaff17035g075846**	KHN75925.1Glutamyl aminopeptidase	629	386<br>545	RFLR<br>RQLR	Sp 0.0055<br>Secp 0.259
**M. javanica_Scaff13529g067570**	XP_014591970.2cytosolic endo-beta-N-acetylglucosaminidase isoform	340	190	RKLR	Sp 0.0011<br>SecP 0.134
**M. javanica_Scaff3885g031723**	XP_026217022.1cytosolic endo-beta-N-acetylglucosaminidase	413	82<br>85	RKLR<br>RGLR	Sp 0.025<br>SecP 0.160
**M. javanica)Scaff30641g097757**	fatty acid desaturase fat-4	265	154	RELR	Sp 0.0008<br>SecP 0.06
